# Effect of Mediterranean Diet Enriched in High Quality Extra Virgin Olive Oil on Oxidative Stress, Inflammation and Gut Microbiota in Obese and Normal Weight Adult Subjects

**DOI:** 10.3389/fphar.2019.01366

**Published:** 2019-11-15

**Authors:** Maria Luisa Eliana Luisi, Laura Lucarini, Barbara Biffi, Elena Rafanelli, Giacomo Pietramellara, Mariaconcetta Durante, Sofia Vidali, Gustavo Provensi, Sara Madiai, Chiara Francesca Gheri, Emanuela Masini, Maria Teresa Ceccherini

**Affiliations:** ^1^Department of Dietology and Clinical Nutrition, IRCCS Don Gnocchi Foundation, Florence, Italy; ^2^Department of Neuroscience, Drug Area and Child Health (NEUROFARBA), University of Florence, Florence, Italy; ^3^Department of Agriculture, Food, Environment and Forestry (DAGRI), University of Florence, Florence, Italy; ^4^Department of Diagnostic Imaging, Molecular Imaging Interventional Radiology and Radiation Therapy, University Hospital Policlinico Tor Vergata, Rome, Italy

**Keywords:** mediterranean diet, high quality-extra virgin olive oil, cytokines, adiponectin, microbiota, lactic acid bacteria, prevention and rehabilitation

## Abstract

**Introduction:** The Mediterranean Diet (MD) is useful in the prevention of overweight, obesity and metabolic disease. High Quality-Extra Virgin Olive Oil (HQ-EVOO), an essential component of this diet, exerts protective effects against chronic diseases. Gut Microbiota (GM), recognized as a key factor in driving metabolic activities, is involved in the regulation of host immunity. Lactic Acid Bacteria (LAB) and their probio-active cellular substances produce beneficial effects in the gastrointestinal tract.

**Materials and Methods:** Eighteen overweight/obese subjects (cases, BMI ≥25 kg/m^2^) and 18 normal weight controls (BMI 18.5–24.9 kg/m^2^) were fed with MD enriched with 40 g/die HQ-EVOO for three months. Feces and blood samples were collected at time 0 (T0) and after three months (T1) for LAB composition, oxidative stress, metabolic and inflammation parameter determinations.

**Results:** Myeloperoxidase and 8-hydroxy-2-deoxyguanosine, markers of inflammation and oxidative stress, were significantly decreased after MD rich in HQ-EVOO both in controls and in cases. Proinflammatory cytokines levels were significantly decreased in cases in comparison to controls, while IL-10 and adiponectin were significantly increased in cases. LAB’s *rpo*B copies/ng of DNA increased 55.6 folds in cases compared to their baseline after MD rich in HQ-EVOO. MD rich in HQ-EVOO increased adiponectin and IL-10 concentration in overweight/obese subjects and decreased oxidative stress and inflammation parameters and at the same time, increased LAB number in GM.

**Discussion:** Our results indicate that MD rich in HQ-EVOO induces an increase of LAB in GM and could have a potential role in the prevention of inflammation.

**Clinical Trial Registration:** www.ClinicalTrials.gov, identifier NCT03441802.

## Introduction

In Systems Medicine, human being is considered as a complex of cells functionally connected, organized and integrated together, with the aim of maintaining a dynamic stability through continuous adapting to the external environment. Overweight and obesity could unsettle this equilibrium predisposing to metabolic syndrome and development of chronic diseases (Poirier et al., 2006).

The pathogenesis for overweight, obesity and some metabolic diseases is multifactorial, involving a complex interaction of genetic, socio-economic, behavioral and environmental aspects. Recently, several studies shed light on a possible correlation between these pathological conditions and alterations in the Gut Microbiota (GM) composition. For instance, evidence indicates beneficial and/or harmful effects on some chronic diseases related with GM differential composition. Furthermore, modification of GM is associated with increased risk of metabolic and immune disorders in animals and humans (Boulangé et al., 2016). High fat and high sugar diets may alter intestinal microbiota independently from differences in host genotype, causing modifications responsible for some of the medical disorders observed in metabolic syndrome (Murphy et al., 2015).

In a recent systematic review and meta-analysis, GM was described as one of the most important risk factors affecting obesity (John et al., 2018), whereby distal GM of obese subjects is different in the relative composition and abundance of bacteria in comparison with non-obese subjects (Turnbaugh et al., 2006).

Native bacteria are mainly acquired at birth and during the first year of life, whereas transient bacteria, the most part of GM, are continuously ingested with food and drinks and their number and composition is modified by life style (Dueñas et al., 2015). Among these microorganisms, Lactic Acid Bacteria (LAB) include a large number of genera such as lactobacilli considered as safe for their hosts, and implicated in the pathogenesis of a number of clinical conditions as diverse as obesity, Cardio Vascular Disease (CVD), inflammatory bowel diseases (Kinross et al., 2011); moreover, complex molecular cross-talk between LAB and host has been demonstrated. LAB produce moonlighting proteins, promote bacterial adhesion to mucosa and stimulate immune cells in response to gut stimuli, and are considered antioxidant and nutraceutical vectors (Pessione, 2012).

Interest for LAB and their potential has recently increased since they were recognized as probiotic agents with an important role in the animal gut ecosystem, as demonstrated in different studies on germ free animals which showed their need of an additional nutritional intake to maintain their ideal weight (Kallus and Brandt, 2012). Previous publications demonstrate that diet consistently influences the intestinal ecosystem and functional capacity of GM of adults and children (Delzenne et al., 2011; Danneskiold-Samsøe et al., 2019).

The Mediterranean Diet (MD) is the gold standard for a healthy nutrition and it is associated with reduced risk of metabolic disease. Its beneficial effect has been attributed not only to macronutrients but also to micronutrients as well as prebiotics, probiotics and other components which can modulate GM. As one of the main constituent of MD, Extra Virgin Olive Oil has a major beneficial role attributed to oleic acid and to polyphenols, that exert antioxidant activity positively influencing several biomarkers of oxidative damage (Hidalgo et al., 2014; Trichopoulou et al., 2014; Gambino et al., 2017; Estruch et al., 2018; Danneskiold-Samsøe et al., 2019)

These polyphenols are directly absorbed or metabolized in the intestine or transformed in active metabolites where they might exert a significant local action in relation to their interaction with the GM. They potentially modulate the oxidative status of intestinal barrier, inflammation and immune response of the host (Deiana et al., 2018).

There is growing evidence that inflammation and oxidative stress are closely related, creating a vicious circle that can aggravate metabolic disease. A low grade inflammation is associated to obesity and adipose tissue releases many active mediators, such as leptin and adiponectin, as well as classical pro-inflammatory cytokines (Feillet-Coudray et al., 2019).

Based on this evidence, the aim of this research was to study, if and how, some parameters of inflammation and oxidative stress and GM’s LAB number copies, change after 3 months of MD rich in High Quality-Extra Virgin Olive Oil (HQ-EVOO) in a cohort of overweight/obese subjects in comparison with normal weight controls. The HQ-EVOO is defined according to the Italian regulation of “high quality” extra virgin olive oil (Annex A) of the Ministry of Agriculture, Food, Forestry and Tourism.

## Materials and Methods

### Enrollment of Subjects

Subjects were recruited at Don Gnocchi Foundation, Florence, Italy. A written informed consent was obtained from each participant. The study was approved by the Ethics Committee of Don Gnocchi Foundation and performed following the Ethical standards of the Declaration of Helsinki, 1964 and its later amendments (Carlson et al., 2004). The number of ClinicalTrials.gov Identifier is NCT03441802.

Inclusion criteria: BMI ≥18.5 kg/m^2^.

Exclusion criteria: Subjects suffering of eating disorders and in recent (1 month) or ongoing antibiotic therapy.

We enrolled 36 individuals, 17 males and 19 females, eligible in the study.

Subjects were divided in two arms according to their BMI: controls had normal weight (BMI between 18.5 and 24.9 kg/m^2^). They were 6 males and 12 females and at enrollment they were following MD, evaluated by a specific score (Sofi et al., 2014). Cases were overweight/obese subjects (BMI ≥25 kg/m^2^). Not all cases, only 11 males and 7 females, at enrollment, followed a MD according to the score criteria.

At enrollment, all the participants to the study received the same procedures and evaluations. At time 0 (T0) all the participants were submitted to anamnestic, anthropometric and dietetic assessments: height was measured on a wall-mounted stadiometer, weight was measured on a leveled platform scale and BMI was calculated as weight (kg)/height (m^2^). The adherence to the MD was evaluated by a specific score from 0 to 18 (0 minimal adherence; 18 greatest adherence) (Sofi et al., 2014). The diet was analyzed using the WinFood database (Medimatica, Teramo, Italy) according to the table of food consumption of the Italian National Institute of Nutrition and to the Food Composition Database for Epidemiologic Study in Italy. The survey items reported the quantities and frequency of food consumption such as cereals, vegetables and fruit, beans, fish, poultry, dairy products, milk, fats and oils, eggs, meat, sweets, salt, alcohol and other beverages (Salvini et al., 1996).

After enrollment all subjects followed a typical MD (55–60% carbohydrates, mainly complex ones, 25–30% polyunsaturated and monounsaturated fats, 15–20% proteins) (Luisi et al., 2015) and cases received a low-calorie MD (Kcal 1,552 ± 160). Both cases and controls utilized 40 g/die of HQ-EVOO for 3 months as the only cooking and dressing fat. HQ-EVOO main constituents are reported in **Table 1** and the analysis were performed by the Valoritalia Laboratories s.r.l., Tavarnelle Val di Pesa, Florence, Italy.

**Table 1:** HQ-EVOO main constituents, analyzed by the Valoritalia Laboratories s.r.l., Tavarnelle Val di Pesa, Florence, Italy.

Main constituents	Method	mg/kg
Oleic acid	Reg. CEE 2568/91	72.84
ß-carotene	MI/C/002 rev 1 20/10/2004	232
Tocopherols (Vit. E)	MI/C/002 rev 3 05/10/2009	197
Polyphenols	MI/C/001 rev 4 05/10/2009	365
Tyrosol	HPLC	2.65
3-hydrossi-Tyrosol	HPLC	2.37
1-acetoxypinoresinol	MI/C/003 rev 4 05/10/2009	48.50

Anthropometric assessments, questionnaires, blood and fecal samples were obtained at T0 and repeated after 3 months of MD rich in HQ-EVOO (T1).

The enrolled individuals followed these drug therapies: one case, affected by type 2 diabetes, received metformin (250 mg × 2/die); four participants had hypertension and were treated with ramipril (5 mg/die).

### Biochemical Analysis

*Ematochemical parameters*: All blood samples were immediately placed on ice and centrifuged at 4°C, after which the plasma was removed. Plasma was stored at −80°C until analysis. Plasma glucose was estimated using a Beckton Dickinson blood sugar assay kit. Ten microliters of plasma was mixed with 90 µl of reagent consisting of glucose oxidase-peroxidase-O-dianisidine. After 10 min, the Optical density (OD) of the complex was measured at 620 nm in an autoanalyzer. Plasma insulin was measured using a human insulin ELISA kit (Novus Biologicals–Bio-Techne Ltd, Abingdon, UK); while plasma C-peptide concentrations were assayed by an ultrasensitive C-peptide ELISA kit (Mercordia AB8, Uppsala, Sweden). The assay was calibrated against International Reference Reagent for C-peptide (IRR-C-peptide, a WHO standard).

*Determination of adiponectin levels:* Adiponectin, an adipocyte-specific protein, which plays a role in the development of insulin resistance, was measured in plasma using a commercially available ELISA kit (Adipo Bioscience, Santa Clara, CA, USA). The assay was carried out according to the manufacturer procedures. The developed color was measured spectrophotometrically using the micro plate reader at 450 nm. Adiponectin concentrations, in µg/ml, were calculated from the standard curve prepared using recombinant human adiponectin standards.

*Determination of 8-hydroxy-2-deoxy-guanosine (8-OHdG):* levels of 8-OH*d*G, a marker of oxidative DNA damage, were measured on plasma (Lucarini et al., 2017). Briefly, frozen plasma samples were added with 1 ml of 10 mmol/l Tris–HCl buffer, pH 8, containing 10 mmol/l EDTA, 10 mmol/l NaCl, and 0.5% SDS, incubated for 1 h at 37°C with 20 µg/ml RNase 1 (Sigma‐Aldrich, Saint Louis, MO, USA) and overnight at 37°C under argon in the presence of 100 µg/ml proteinase K (Sigma‐Aldrich). The mixture was extracted with chloroform/isoamyl alcohol (10/2 v/v). DNA was precipitated from the aqueous phase with 0.2 volumes of 10 mmol/l ammonium acetate, solubilized in 200 µl acetate buffer, pH 5.3 and denatured at 90°C for 3 min. The extract was then supplemented with 10 IU of P1 nuclease (Sigma‐Aldrich) in 10 µl and incubated for 1 h at 37°C with 5 IU of alkaline phosphatase (Sigma‐Aldrich) in 0.4 mol/l phosphate buffer, pH 8.8. All of the procedures were performed in the dark under argon. The mixture was filtered by an Amicon Micropure‐EZ filter (Merck‐Millipore, Milan, Italy) and 50 µl of each sample was used for 8‐OH*d*G determination using a ELISA kit (JalCA, Shizuoka, Japan), following the instructions provided by the manufacturer. The absorbance of the chromogenic product was measured at 450 nm and expressed as ng/mg of DNA. The results were calculated from a standard curve based of 8‐OH*d*G solution. The values are expressed as ng 8‐OH*d*G/ng total DNA.

*Determination of malonyldialdehyde (MDA)*: the measurement of lipid peroxidation in plasma samples was based on the reaction of MDA, the end product of the process, with 2-thiobarbituric acid (TBA) to form a chromophore absorbing at 532 nm. The reaction mixture (0.4 ml of sample; 0.2 ml of 2 mM chlortetramethoxypropane; 0.2 ml of 8.1% sodium dodecylsulphate; 1.5 ml of 20% acetic acid and 1.5 ml of aqueous solution of TBA up to 4 ml with distilled water) was heated to 95°C for 30 min and then 1 ml of N-butanol and pyridine (15:1 v/v) was added to plasma samples. The absorbance of the organic phase was measured at 532 nm (Mullane et al., 1985). The values were expressed as nanomoles of TBA-reactive substances (MDA equivalent) mg^−1^ of protein, determined with the Bradford method (Bradford, 1976) over an albumin standard curve.

*Determination of myeloperoxidase (MPO) activity*: myeloperoxidase, a reliable marker of leukocyte activation, was determined as described in the literature (Masini et al., 2002) with some modifications: 100 ml of plasma was allowed to react with a solution 1.6 mM of tetramethylbenzidine and 0.1 mM H_2_O_2_. The rate of change in absorbance was measured spectrophotometrically at 650 nm. MPO activity was defined as the quantity of enzyme degrading 1 *µ*mol of peroxide per minute at 37°C and was expressed in mU per mg of protein, determined with the Bradford method (Bradford, 1976) over an albumin standard curve.

*Determination of the cytokines:* the levels of two pro‐inflammatory cytokines, interleukin-6 (IL‐6) and tumor necrosis factor-α (TNF-α) and anti-inflammatory interleukin-10 (IL-10) were measured on aliquots (50 µl) of plasma by using the Flow Cytomix assay (Bender Medsystems GmbH, Vienna, Austria), following the protocol provided by the manufacturer. Fluorescence was read with a cytofluorimeter (CyFlow^®^ Space, Partec, Germany). Values are expressed as pg/µg of total proteins determined over an albumin standard curve (Bradford, 1976).

### Monitoring of Gut Microbiota: DNA Extraction and Quantification

Total DNA (Agnelli et al., 2004) was extracted from fecal samples by following the QIAamp DNA Stool Mini Kit instructions (Qiagen) and quantified with a Qubit^®^ 2.0 fluorometer (Invitrogen, USA). Molecular weight and fragment length of DNA were checked on 1.5% agarose gel; the yield was calculated as µg DNAg^−1^ feces. Quantitative PCR (qPCR) was conducted using the specific primers *rpo*B1, *rpo*B1o and *rpo*B2 for the RNA-polymerase β subunit encoding gene, a valuable alternative as present in single copy per genome (Dahllöf et al., 2000; Adékambi et al., 2008; Větrovský and Baldrian, 2013), that generate amplicons of 250-bp (Renouf et al., 2006), on 10 ng DNA for all the samples; negative control contained only ddH_2_O. Reactions were performed in a CFX Connect 96 apparatus (BioRad, Hercules, CA, USA) and the results were analyzed by the manufacturer’s software. Amplification was carried out in a 25 µl final volume containing: 7.5 µM of each primer, 2.5 µg of BSA, 1X iTaq^™^ Universal SYBR^®^ Green Supermix, sterile ddH_2_O to reach the appropriate volume. Amplification was performed in 96-well micro titer plates (BioRad). The program cycle was: 95°C 3 min followed by 35 cycles of 95°C 1 min, 45°C 1 min and 72°C 1 min. After that, a melting curve program was run for which measurements were made at 0.5°C temperature increments every 10 s within a range of 60–100°C. A *rpo*B amplified and purified fragment (from a mix of LABs of a commercial probiotic formulation—Lactoflorene^®^ Plus, Montefarmaco OTC, (Soldi et al., 2019)) was used as standard.

The standard curve was developed by plotting the logarithm of known concentrations (tenfold dilution series in triplicate from 6.5 × 10^-1^ to 6.5 × 10^−5^ in 25 µl reaction) of the *rpo*B fragment against the threshold Cycle (Ct) values (Ceccherini et al., 2003). The qPCR standard curve had an R^2^ of 0.99–0.97 and an efficiency >85%. Three replicates were carried out for each sample for the two interval times: T0 and T1.

### Statistical Analysis

Sample size calculation was performed to assess the minimum number of participants to be included in the study (website: http://hedwig.mgh.harvard.edu/sample_size/size.html) (Schoenfeld, 2001). It was calculated considering that 80% was the probability that the study revealed a difference of MPO levels between the two groups (cases and controls at T0) with a significance level of 0.05, assuming that the set power was 0.8. The minimum sample size for this study was 18 subjects.

Data were reported as mean values of individual average measures of each subject. Delta (Δ) is the variation between T0 and T1. Delta values were calculated by subtracting the final value at 3 months of MD from the corresponding initial value at baseline. Significance of differences among the groups was assessed by *t*-test or repeated measures two-way analysis of variance followed by Bonferroni’s post-hoc test using GraphPad Prism 4.03 statistical software, when appropriated, and QuickCalcs (GraphPad Software, Inc, La Jolla, CA).

## Results

Subjects were divided in two arms according to their BMI: controls (6 males and 12 females non-obese subjects with a BMI at T0 21.6 ± 2.6 and at T1 21.7 ± 2.4 kg/m^2^, mean age 41.4 ± 14.42), and cases (11 males and 7 females overweight/obese subjects with a BMI at T0 30.152 ± 4.8 and at T1 28.7 ±4.1 kg/m^2^, mean age 52.1 ± 13.04). The demographic and clinical characteristics of the studied population are described in **Table 2**.

**Table 2:** Demographics and clinical characteristics of the studied population.

Characteristics	Controls n.18	Cases n.18
Age (years)	41.4 ± 14.42 (range 24–71)	52.1± 13.04 (range 20–61)*
Gender (male/female)	M 6 + F 12	M 11 + F 7
Type 2 Diabetes (*)	0/18	1/18
Arterial Hypertension (**)	1/18	3/18
Smokers (more than 5 cigarettes/day)	1/18	4/18

*Diagnosis of type 2 diabetes: fasting plasma glucose >126 mg/dl (7.0 mmol/l); according to American Diabetes Association Standards of Medical Care in Diabetes, 2017 (American Diabetes Association, 2017) and (**) diagnosis of arterial hypertension: >140/90 mmHg, according to the Task Force for the Management of Arterial Hypertension of the European Society of Hypertension and the European Society of Cardiology (Mancia et al., 2013). Values are mean ± SD for n = 18 subjects/group.

*p < 0.05, t-test was used for the analysis.

### Anthropometric and Hematochemical Parameters

Body weight and BMI were significantly different at the same time point between controls and cases (controls T0 *vs* cases T0 and controls T1 *vs* cases T1). Moreover, cases at T1 showed a significant decrease in BMI compared to T0. The Δ T1 − T0 confirmed that these differences were significant in cases (**Table 3**).

**Table 3:** Anthropometric and hematochemical parameters of the studied population.

Parameters	Controls		Cases		ΔT_1_ − T_0_	
	T_0_	T_1_	T_0_	T_1_	Controls	Cases
Weight (kg)	60.9 ± 3.1	61.2 ± 3.0	82.7 ± 3.4***	79.2 ± 3.2***	0.4 ± 0.4	−3.5 ± 1.2^§^
BMI (kg/m^2^)	21.6 ± 0.6	21.7 ± 0.6	30.2 ± 1.0***	28.8 ± 0.9***^,##^	0.1 ± 0.1	−1.3 ± 0.4^§^
Total Cholesterol (mg/dl)	201.8 ± 9.9	201.2 ± 14.2	195.5 ± 7.6	197.6 ± 9.3	1.4 ± 5.9	2.0 ± 6.2
HDL Cholesterol (mg/dl)	51.0 ± 5.0	55.3 ± 5.2	58.5 ± 4.0	61.6 ± 3.8	4.3 ± 3.1	3.1 ± 2.2
LDL Cholesterol (mg/dl)	130.3 ± 8.1	125.8 ± 10.8	117.3 ± 7.4	117.8 ± 8.8	−4.5 ± 4.7	0.5 ± 5.0
Triglycerides (mg/dl)	102.5 ± 12.8	93.9 ± 7.7	98.9 ± 7.4	90.9 ± 6.0	−8.7 ± 17.4	−8.0 ± 8.2
Fasting glucose (mg/dl)	96.6 ± 3.9	92.0 ± 4.9	88.7 ± 1.9	91.0 ± 2.0	−4.7 ± 3.3	2.2 ± 1.4^§^
Insulin (µU/ml)	22.9 ± 2.3	21.7 ± 1.8	29.0 ± 1.4*	26.9 ± 1.4^#^	−1.2 ± 0.9	−2.1 ± 0.5
C-peptide (ng/ml)	1.7 ± 0.1	1.6 ± 0.1	2.2 ± 0.1**	1.8 ± 0.1	−0.2 ± 0.03	−0.4 ± 0.1

*P < 0.05, **P < 0.01 and ***P < 0.001 vs control group at same time point (ex. Control T_0_ x Case T_0 _).

^#^P < 0.05, ^##^P < 0.01 and ^###^ P <0.001 vs T_0_ in the same group (ex. Control T_0_ x Control T_1 _).

Both Two-Way ANOVA and Bonferroni’s MCT.

^§^P < 0.05 vs controls. Unpaired t-test.

Total, HDL, LDL cholesterol and triglycerides did not show any statistical difference between cases and controls at T0 and T1. The Δ T1 − T0 of fasting glucose was significant in cases. Insulin and C-peptide were higher in cases both at T0 and T1 in comparison to controls (**Table 3**).

### Oxidative Stress and Inflammatory Markers

**Figure 1:**
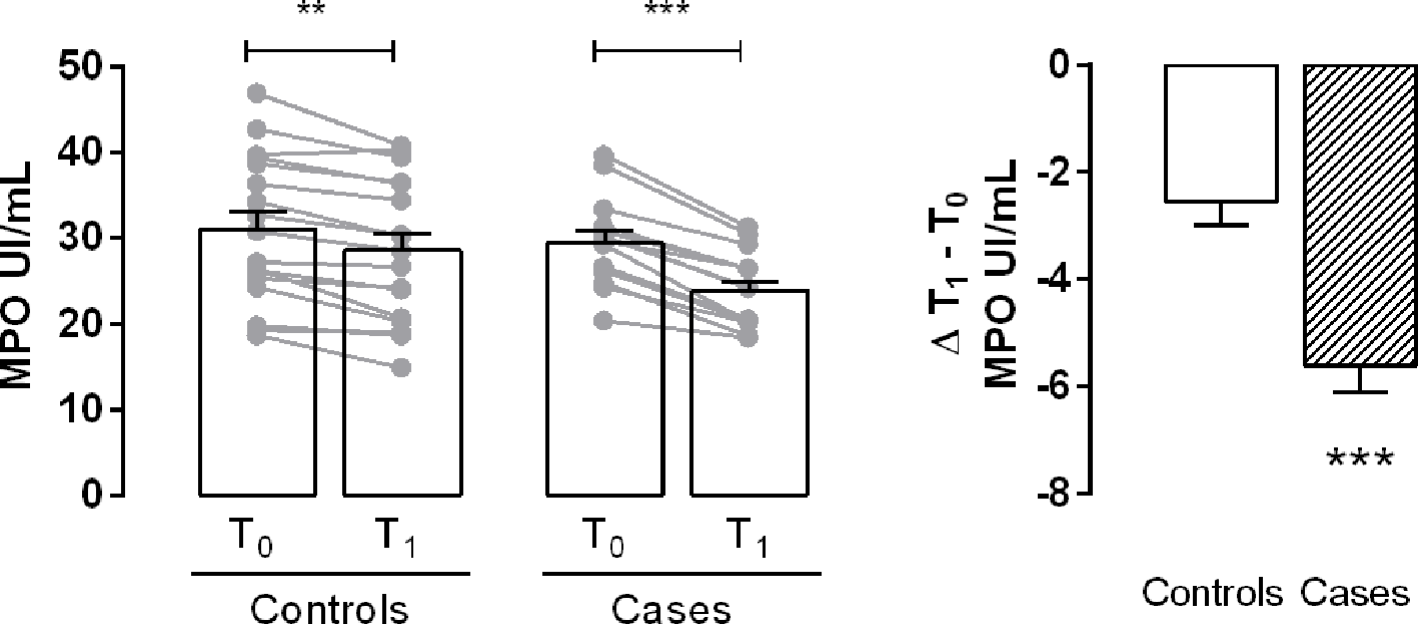
Evaluation of myeloperoxidase (MPO) activity. Bar graph shows the plasma levels of the enzyme in controls and cases at T0 and T1. Delta (Δ) values are calculated by subtracting the final value at 3 months from the corresponding initial value at baseline. Values are mean ± S.E.M. for n = 18 subjects/group. **p < 0.01, ***p < 0.001 *vs* T0 and *vs* controls. Two-way ANOVA followed by Bonferroni’s post-hoc test was used for the analysis of differences among the groups; *t*-test was used for the variation analysis (Δ T1 − T0).

**Figure 2:**
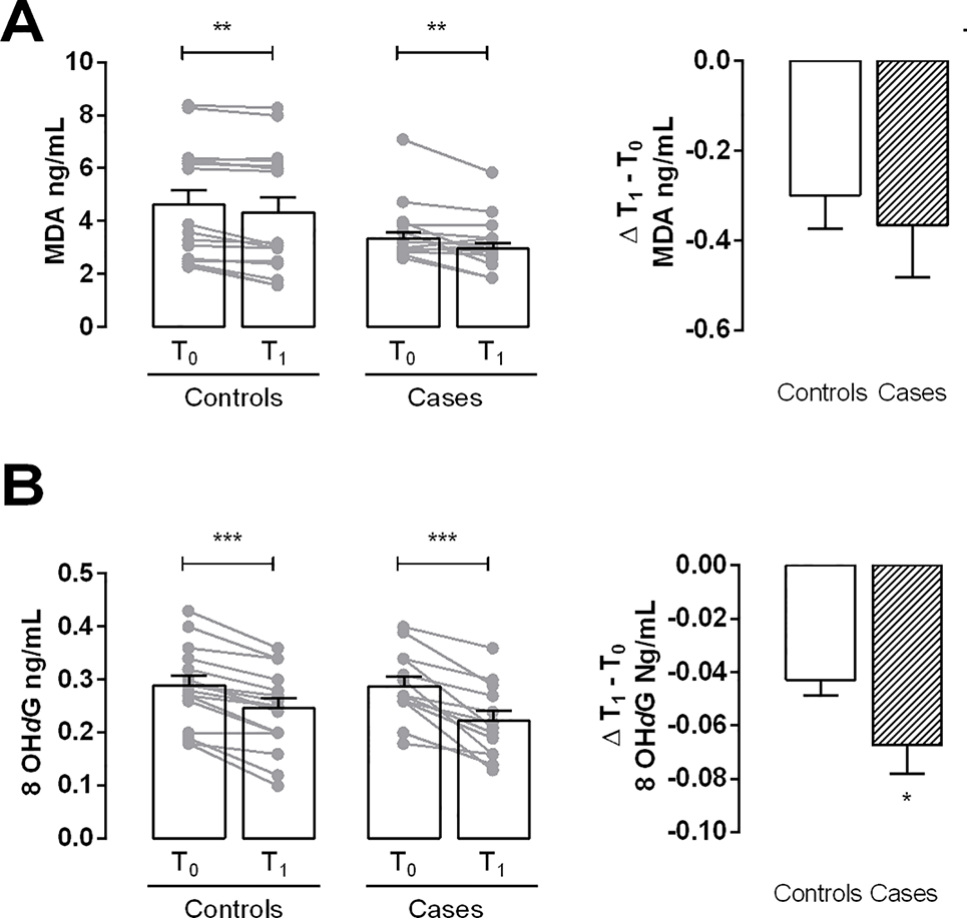
Evaluation of oxidative stress markers. **(A)** Bar graph shows the levels of malonyldialdehyde (MDA) in controls and cases at T0 and T1. **(B)** Bar graph shows the levels of 8-hydroxy-deoxyguanosine (8-OH*d*G) in controls and cases at T0 and T1. Delta (Δ) values are calculated by subtracting the final value at 3 months from the corresponding initial value at baseline. Values are mean ± S.E.M. for n = 18 subjects/group. *p < 0.05 *vs* control,**p < 0.01 *vs* T0; ***p < 0.001 *vs* T0. Two-way ANOVA followed by Bonferroni’s post-hoc test was used for the analysis of differences among the groups; *t*-test was used for the variation analysis (Δ T1 − T0).

Myeloperoxidase levels were significantly lower at time T1 than at time T0 in both controls and cases. The Δ T1 − T0 indicated that this difference was significant in cases (**Figure 1**). The amount of MDA, a marker of lipid peroxidation, was significantly lower at T1 both in controls and cases (**Figure 2A**). The production of 8-OH*d*G, a reliable marker of DNA oxidative stress, measured at T1, both in controls and in cases, was significantly decreased in comparison to the values at T0, mostly in cases (**Figure 2B**).

**Figure 3:**
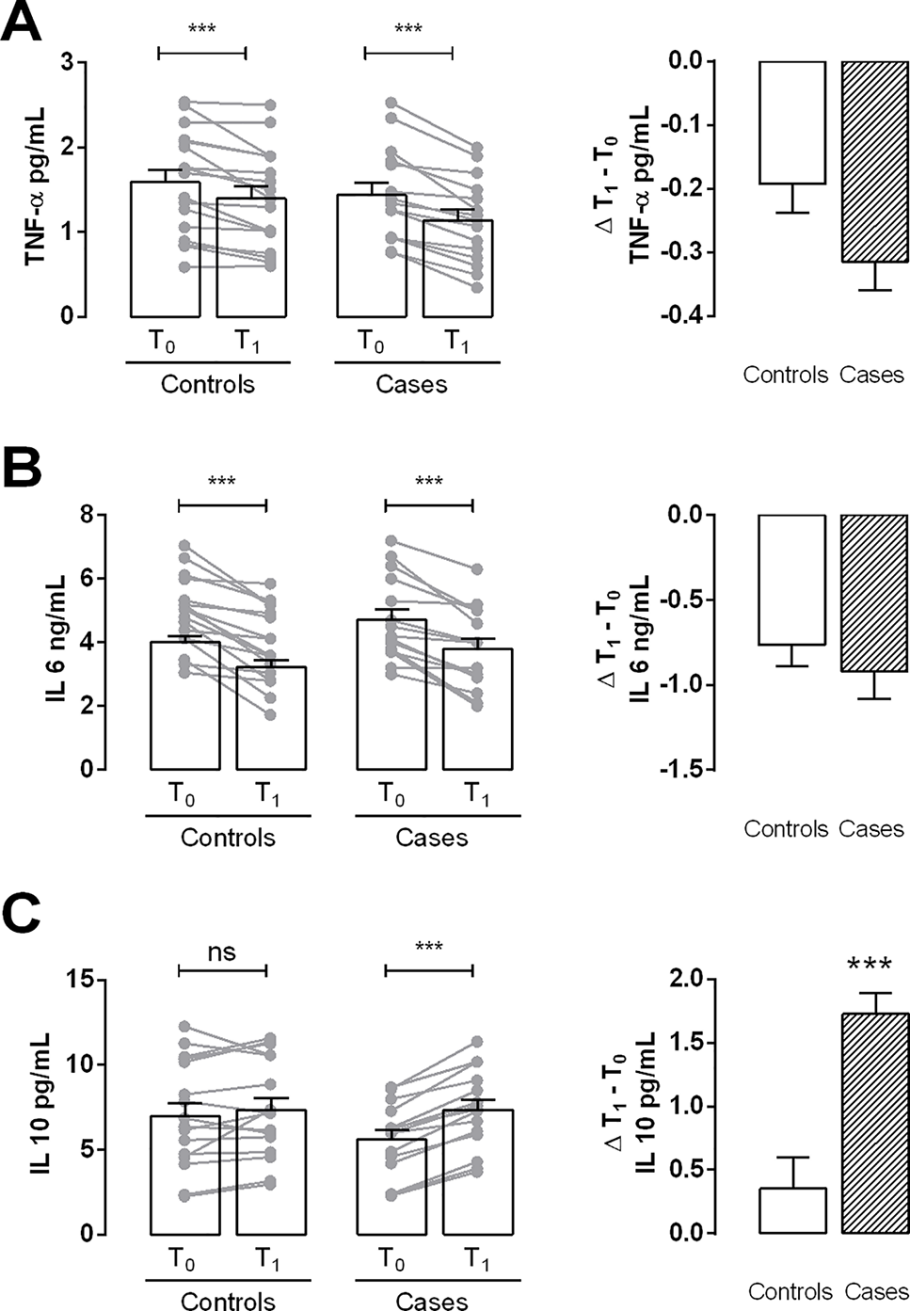
Determination of inflammation parameters. **(A** and **B)** Analysis of pro-inflammatory cytokines TNF-α and IL-6 content (respectively) in plasma samples of controls and cases at T0 and T1. **(C)** Bar graph shows the plasma levels of the anti-inflammatory cytokine IL-10 in controls and cases at T0 and T1. Values are mean ± S.E.M. for n = 18 subjects/group. ***p < 0.001 *vs* T0 and *vs* control. ns = not significant. Two-way ANOVA followed by Bonferroni’s post-hoc test was used for the analysis of differences among the groups; *t*-test was used for the variation analysis (Δ T1 − T0).

The levels of TNF-α and IL-6 showed a decreasing trend from T0 to T1, both in controls and cases, although these differences were not significant (**Figures 3A, B**). Interestingly, in cases, the level of IL-10, an anti-inflammatory cytokine, was significantly higher at T1 than at T0; no statistical significant differences were found in controls (**Figure 3C**).

### Adiponectin Levels

**Figure 4:**
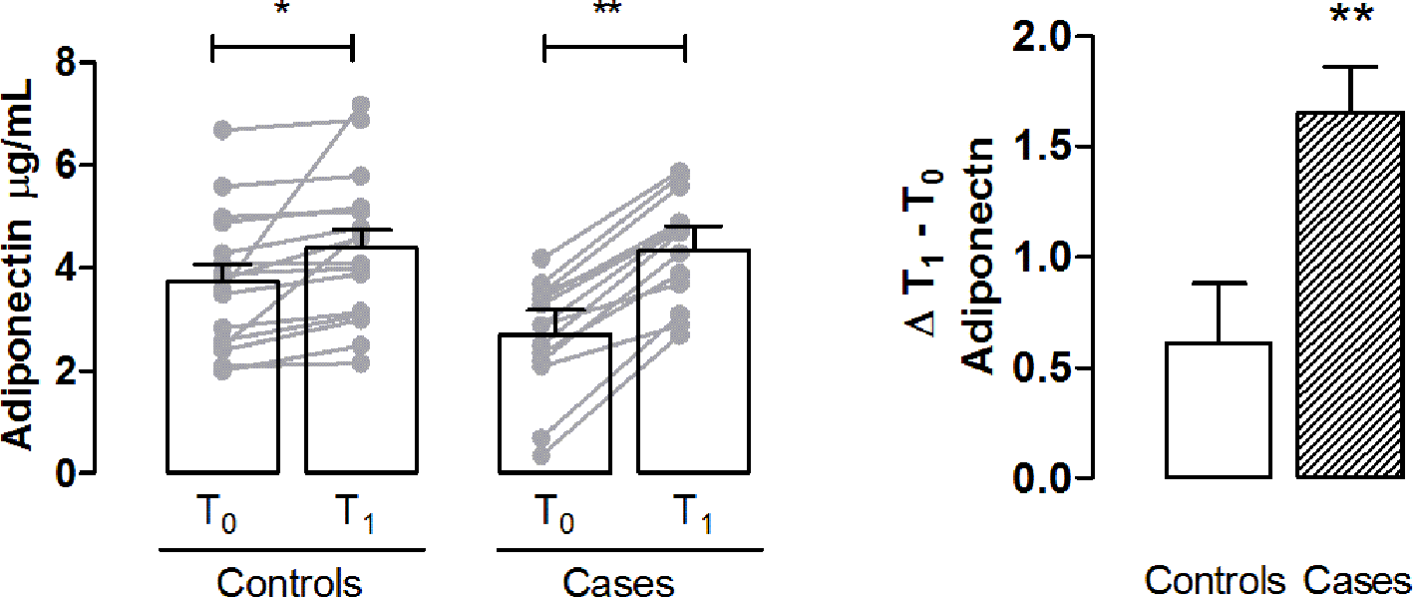
Determination of plasma adiponectin as a marker of metabolic dysregulation. Bar graph shows the plasma levels of adiponectin in controls and cases T0 and T1. Delta (Δ) values are calculated by subtracting the final value at 3 months from the corresponding initial value at baseline. Values are mean ± S.E.M. for n = 18 subjects/group. *p < 0.05, **p < 0.01 *vs* T0 and *vs* control. Two-way ANOVA followed by Bonferroni’s post-hoc test was used for the analysis of differences among the groups; *t*-test was used for the variation analysis (Δ T1 − T0).

At T0, the control group showed higher mean plasma adiponectin levels compared to those of cases. For both groups there was a statistically significant increase in adiponectin levels after MD rich in HQ-EVOO. T1 − T0 Δ resulted to be 0.6 ± 0.26 in controls while 1.6 ± 0.2 in cases (**Figure 4**).

### Monitoring of the Gut Microbiota: Fecal Biomass DNA Extraction and LAB Quantification

In our study, the yield of dsDNA per gram of feces, extracted from all the samples, grouped as controls and cases at times T0 and T1, was not significantly different, neither between cases nor between times (controls T0/T1, cases T0/T1, controls T0/cases T0, controls T1/cases T1, data not shown). We can be assumed that there was no modification measurable by Qubit.

**Figure 5:**
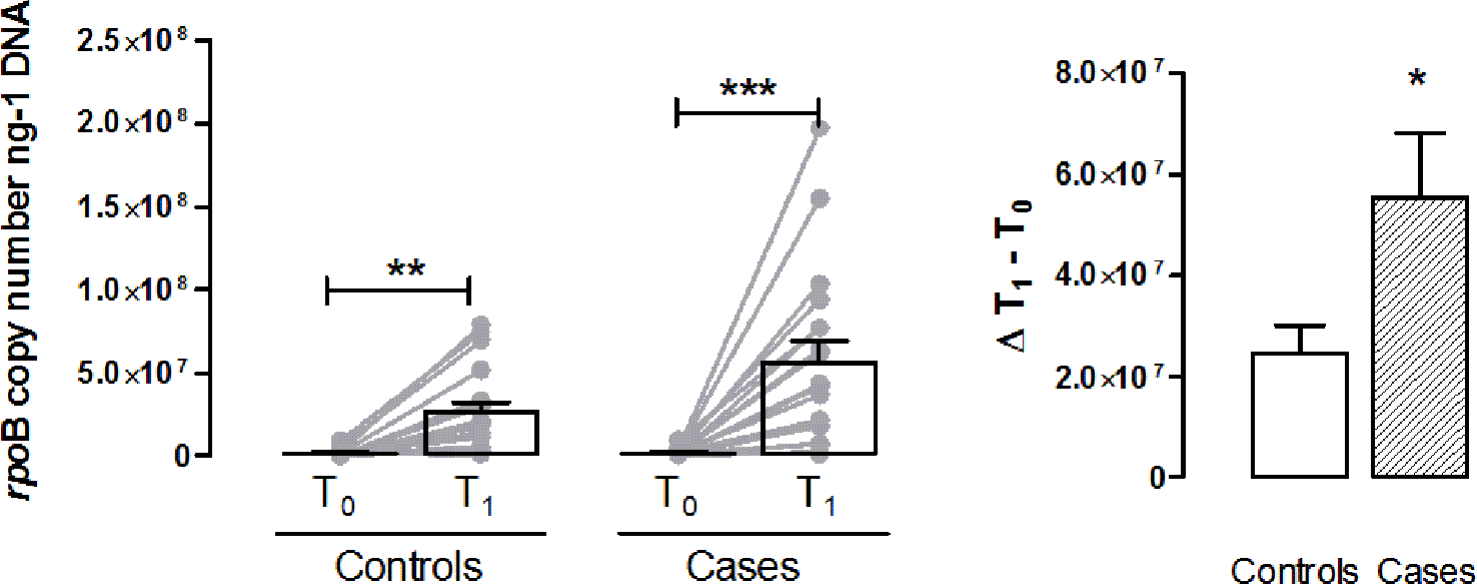
LAB’s *rpo*B sequences quantification. Bar graph shows *rpo*B copy number/ng of DNA in controls and cases at T0 and T1. Values are mean ± S.E.M. for n = 18 subjects. *p < 0.05, **p < 0.01 *vs* T0 controls and ***p < 0.001 *vs* T0 cases. Two-way ANOVA followed by Bonferroni’s post-hoc test was used for the analysis of differences among the groups; *t*-test was used for the variation analysis (Δ T1 − T0).

Regarding the specific LAB’s *rpo*B sequences quantified by qPCR, significant differences were assessed between controls and cases as a function of time. The *rpo*B copy number per ng of template DNA was significantly increased in cases and controls at T1; while, at T0 the *rpo*B copies between controls and cases were not significantly different (**Figure 5**). In particular, the amount of *rpo*B copies in controls at T1 increased 11.4 times than those in the same samples at T0, while in cases at T1 *rpo*B amount increased 55.6 times than those at T0. Moreover these results showed that the *rpo*B amount in cases at T1 were significantly higher than the ones in controls.

## Discussion

Obesity is a growing problem with epidemic proportions worldwide, has a great effect on diabetes and CVD risk and induces the production of several cytokines and inflammatory markers that contribute to CVD outcome in overweight and obese people (Van Gaal et al., 2006). A healthy MD was shown to be cardio-protective and has been linked to a number of health benefits. In this context, there is increasing evidence of health benefits of HQ-EVOO, the main fat in MD (Trichopoulou et al., 2014; Martín-Peláez et al., 2017) and recent evidences suggest that extra virgin olive oil polyphenols health benefits, may be also related to the modulation of GM balance (Hervert-Hernández and Goñi, 2011).

The results of the present study suggest that HQ-EVOO is an important component of the anti-inflammatory action of MD, since MPO (a reliable marker of inflammation and endothelial dysfunction), 8-OH*d*G (a pro-mutagenic oxidative adduct in the DNA of all tissues and organs), TNFα and IL-6 (cytokines that impair insulin receptor signaling and activate inflammatory cascade) are significantly decreased in both cases and controls subjects. Plasma adiponectin, which induces IL-10 mRNA expression in human macrophages and IL-10 itself, increased at T1, suggesting a further protective role of HQ-EVOO rich in polyphenols. These compounds, such as oleuropein, have shown antioxidant, anti-inflammatory and anti-thrombotic properties, improving endothelial function (Oliveras-López et al., 2014). In particular, oleuropein improves postprandial glycaemia in healthy subjects *via* an oxidative stress‐mediated mechanism (Carnevale et al., 2018). Moreover, our results suggest that gut LAB promptly responded increasing in number after the introduction of HQ-EVOO rich in polyphenols as the main fat component of the MD. Owing to its many roles in human health, there is great interest in deciphering the principles that govern an individual’s GM. Anyway, the inter-relationship between our dietary habits and the structure of our GM is still poorly understood. Preliminary data suggest that in mice dietary saturated fats, rather than unsaturated fats, indirectly modulate GM composition and may contribute to the development of metabolic syndrome (de Wit et al., 2012). In this regard, HQ-EVOO was rarely used as a monounsaturated fat for studies on its effects on human obesity, hepatic steatosis or GM composition.

The phenolic fraction of HQ-EVOO, besides oleic acid, also acts as promoting factor of growth or survival for beneficial gut bacteria, mainly *Lactobacillus* strains, and inhibiting the proliferation of some pathogenic bacteria (Martín-Peláez et al., 2017).

The use of the *rpo*B gene in qPCR, the most quantitative and reliable tool to determine bacterial concentrations in environmental and clinical samples, allowed to refer directly to the number of LAB because this gene is present in single copy in the bacterial genome. In this work, the gut biomass as a whole, calculated by the dsDNA extracted from feces, did not significantly differ between cases and controls during the experiment. However, what caught our attention was, instead, the amount of *rpo*B sequences that is the significant increment of the number of LAB at the end of the monitoring diet period, in both cases and controls. At T0 the amount of LAB was not significantly different between controls and cases, but at T1, the presence of these bacterial population increased significantly in both groups and particularly in cases and in a relatively short period of time.

Although gender and age variations influence GM (Spychala et al., 2018; Santos-Marcos et al., 2019), in the present study these stratification analyses were not performed for the reduced number of the enrolled subjects; however, the higher effect of MD rich in HQ-EVOO, on anti-inflammatory cytokines and LAB, observed in overweight/obese than in normal weight subjects, indicates the validity of our hypothesis. Further investigation is needed to clarify the etiopathogenetic mechanism.

In conclusion, obesity, metabolic disease and inflammation are considered to be nutrition-related disorders and recent evidence indicates that GM plays a major role both in disease development and in wellness. Dietary polyphenols, such as those present in High Quality-Extra Virgin Olive Oil, are substrates that seem to contribute to the maintenance of GM, mainly *Lactobacillus* strains, and thus, exerting prebiotic actions. There are still few human trials that have been carried out to test the efficacy of MD as anti-obesity and anti-inflammatory treatment by inducing a modification of Lactic Acid Bacteria. Our results, supporting the role of GM as an important element in the regulation of energy homeostasis, contribute to unveil the potential role of the MD focused on HQ-EVOO rich in polyphenols use, as a possible diet-related intervention, for decreasing inflammation and oxidative stress both in normal and overweight/obese subjects in prevention and rehabilitation.

## Data Availability Statement

The datasets generated for this study are available on request to the corresponding author.

## Ethics Statement

The study was approved by the Ethics Committee of Don Gnocchi Foundation and performed following the Ethical standards of the Declaration of Helsinki, 1964 and its later amendments (Carlson et al., 2004). The number of ClinicalTrials.gov Identifier is NCT03441802. The patients/participants provided their written informed consent to participate in this study.

## Author Contributions

ML: designed research, provided essential reagents and materials, wrote the manuscript, and had primary responsibility for final content. EM: designed research, provided essential reagents and materials, and wrote the manuscript. ER designed research and enrolled subjects. BB, SM, and CG: selected and followed subjects participating to the study and prepared the samples for the biochemical determinations. MC and GPi: designed and conducted research, analyzed molecular data, performed statistical analysis, and wrote paper. LL and MD: conducted the research. GPr: performed statistical analysis and the evaluation of the results. SV wrote the manuscript. All authors read and approved the final manuscript.

## Conflict of Interest

The authors declare that the research was conducted in the absence of any commercial or financial relationships that could be construed as a potential conflict of interest.
